# Herpes Simplex Virus Esophagitis in an Immunocompetent Patient: A Case Report

**DOI:** 10.7759/cureus.68755

**Published:** 2024-09-05

**Authors:** Vishal Padwale, Vijendra Kirnake, Ravi Daswani, Anusha Gupta, Kamlesh Taori, Virendra Bhad

**Affiliations:** 1 Department of Medical Gastroenterology, Jawaharlal Nehru Medical College, Datta Meghe Institute of Higher Education & Research (DMIHER), Wardha, IND; 2 Department of Gastroenterology, Jawaharlal Nehru Medical College, Datta Meghe Institute of Medical Sciences (Deemed to be University), Wardha, IND; 3 Department of Gastroenterology, Datta Meghe Institute of Higher Education & Research (DMIHER), Wardha, IND; 4 Department of Gastroenterology and Hepatology, Jawaharlal Nehru Medical College, Datta Meghe Institute of Higher Education & Research (DMIHER), Wardha, IND

**Keywords:** endoscopy, gastroesophageal reflux disease (gerd), histopatholgy, hsv esophagitis, immunocompetent host

## Abstract

Even though it is rare, herpes simplex virus (HSV) esophagitis has a significant adverse impact on immunocompromised people, such as those with HIV, cancer patients receiving chemotherapy or radiation to the neck, and recipients of transplants receiving immunosuppressive treatments. This makes a high level of clinical suspicion necessary for a precise diagnosis and successful treatment. Although rare, its occurrence in immunocompetent patients poses unique challenges for diagnosis and therapy.

A 68-year-old woman with HSV esophagitis presented with severe symptoms, including odynophagia and hematemesis. Endoscopy revealed “volcano-like” ulcers; after confirmation of HSV-1 infection, treatment with acyclovir, esomeprazole, and sucralfate led to symptom resolution within a week and complete healing in three months.

This case underscores the importance of considering HSV esophagitis in the differential diagnosis of esophageal ulcers in immunocompetent patients, emphasizing the need for early diagnosis and antiviral therapy for effective treatment.

## Introduction

Herpes simplex virus (HSV) esophagitis is an infrequent and often underappreciated modern-day healthcare problem that occurs predominantly in immunocompromised patients such as individuals with HIV infection, malignancies receiving chemotherapy or radiation therapy to the neck region, and hematopoietic stem cell transplant recipients taking immunosuppressive therapies. These weakened immune defenses allow a common pathogen, HSV, to reactivate and infect the esophageal mucosa in these patients. Symptoms and signs include odynophagia, dysphagia, or chest pain with a high index of suspicion that aids in diagnosis to manage these banging courses effectively [1].

Although an established etiology in the immunocompromised, HSV esophagitis is less infrequently seen and understood in terms of its true incidence and clinical course when presented even among otherwise healthy hosts. Immunocompetent patients have their immune system protecting them against extensive mucosal disease and frequent recurrences of HSV. Nevertheless, HSV esophagitis can develop in such patients, suggesting that additional factors helped to trigger the esophageal infection despite low immune impairment [2-4].

This case report details the presentation of HSV esophagitis in an elderly female synchronously outside any observed immunocompromising state. Her presentation adds to our understanding of the disease spectrum and triggers, which we now know can include HSV esophagitis in older, otherwise immunocompetent hosts. The clinical context of HSV esophagitis in immunocompetent patients is important because it emphasizes the need for medical professionals to think when diagnosing esophageal symptoms in this patient population. Chest pain, dysphagia, and odynophagia are symptoms frequently linked to more common illnesses such as peptic ulcer disease, esophageal candidiasis, and gastroesophageal reflux disease (GERD). As a result, it is simple to miss the diagnosis of HSV esophagitis, which might result in improper or delayed therapy [5].

## Case presentation

A 68-year-old woman who had a history of heartburn and dysphagia arrived at the Shalinitai Meghe Superspeciality Center in February 2024 complaining of intense burning pain that traveled from her throat to her stomach, along with nausea and bright red blood streaks in her vomit. She stated that she had experienced odynophagia, or uncomfortable swallowing for the previous five days. Her medical history included controlled hypertension. Upon physical inspection, she looked averagely built and well-nourished. No features are suggestive of herpes infections such as oral ulcerations, angular stomatitis, conjunctivitis, or keratitis. Her vital signs were normal. The results of laboratory testing, such as the complete blood count, random blood sugar, liver function test, renal function test, glycosylated hemoglobin (HbA1c), hepatitis B surface antigen (HbsAg), anti-hepatitis C virus (HCV) antibody, and HIV were within normal ranges.

**Figure 1:**
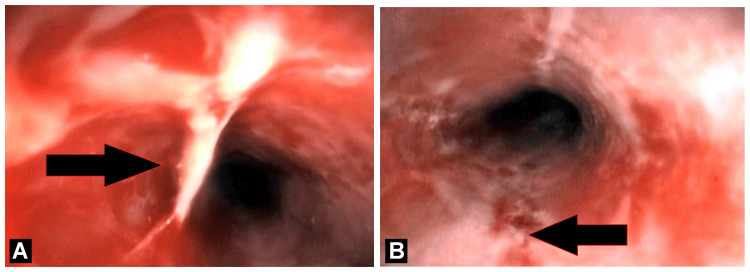
Endoscopic finding of HSV esophagitis. (A) Esophageal ulcers with raised mucosa - “volcano-like appearance”. (B) Deep esophageal ulceration. HSV - Herpes simplex virus

The first endoscopic image reveals multiple well-defined ulcerations within the esophageal mucosa, each exhibiting raised, erythematous borders and central depressions, often described as having a “volcano-like” appearance present in mid esophagus approximately 24 cm from incisors teeth. The surrounding mucosa appears relatively normal, a distinguishing feature of HSV-related lesions. The second image further illustrates similar but diffuse, extensive ulcerative lesions along the esophageal lining, with the mucosa showing areas of erythema and inflammation interspersed with discrete, well-circumscribed ulcers in Mid esophagus approximately 27 cm away from incisor teeth. This pattern is consistent with HSV esophagitis, where the viral cytopathic effect results in distinct ulcer formations (Figures 1A, 1B).

**Figure 2:**
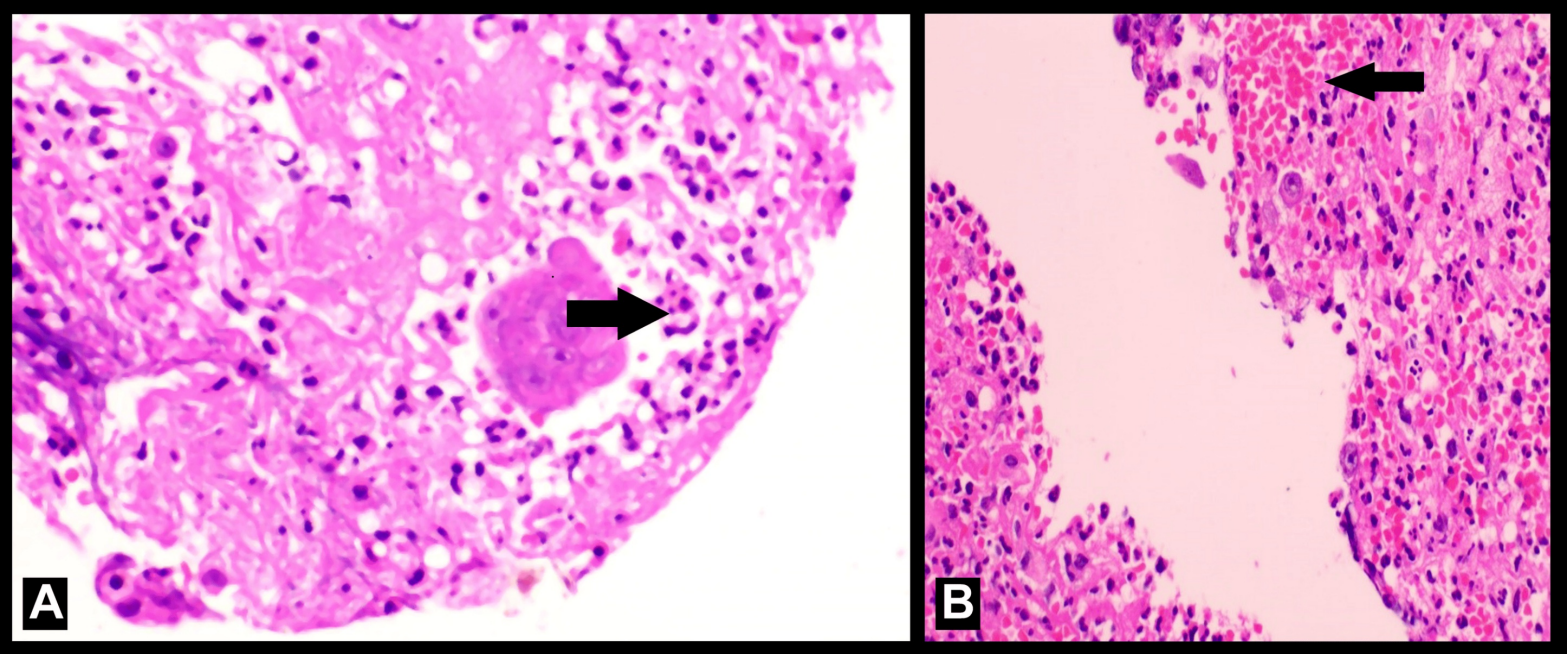
Histopathology image of endoscopic esophageal biopsy. (A) Nuclear enlargement and occasional giant cell. (B) Ground glass nuclei with dense inflammatory infiltrate.

Biopsies were taken from the ulcer margins in the esophagus under endoscopic guidance for histopathological examination and immunostaining. The histological examination showed multinucleation, nuclear molding, nuclear enlargement, occasional giant cell, and ground glass nuclei with dense inflammatory infiltrate and margination, consistent with HSV infection (Figures 2A, 2B).

Serological tests for HSV, including HSV 1 and HSV 2, Immunoglobulin M (IgM) and Immunoglobulin G (IgG), were performed. Initial results were negative for HSV-1 IgM/IgG, but follow-up testing two months later showed seroconversion with positive HSV-1 IgG, confirming recent infection.

The patient was treated with oral acyclovir (400 mg, three times daily for 10 days), a proton pump inhibitor (Esomeprazole), and sucralfate to manage esophageal symptoms and promote mucosal healing. Her symptoms significantly improved within a week of starting antiviral therapy, and a follow-up Endoscopy at three months showed complete resolution of esophageal ulcers.

## Discussion

The importance of recognizing this esophagitis in immunocompetent patients is underscored by the potential benefits of timely antiviral therapy. Antiviral therapy can greatly speed up symptom resolution and increase patient comfort, even though the illness may heal on its own in one to two weeks. Furthermore, knowing the causes and risk factors of HSV esophagitis in this population can help create care protocols and preventative measures. Research on the pathophysiology of HSV esophagitis in immunocompetent people is still ongoing. Numerous theories have been put out, such as temporary immunosuppression brought on by stress (either physical or mental), coexisting infections, or the short-term use of immunosuppressive drugs (e.g., corticosteroids). Furthermore, immune function might deteriorate with age, making older persons more susceptible to infections usually associated with immunocompromised patients [6].

In this instance, her advanced age may have contributed to her vulnerability to Herpes esophagitis even though she was immunocompetent. Immunosenescence, or age-related changes in immune function, is common in the elderly and can make it more difficult for the body to combat latent viral infections successfully. This case report aims to investigate these variables in more detail and offer insights into the manifestation of HSV esophagitis in hosts who appear to be immunocompetent. The histopathological study, endoscopic inspection, and clinical assessment are all used in the diagnostic process for HSV esophagitis [7].

An essential diagnostic technique for HSV esophagitis is endoscopy. A “volcano-like” appearance and numerous small, well-circumscribed ulcers, frequently dispersed over the mid and distal esophagus, are prominent observations. These ulcers can be distinguished from other types of esophagitis by having normal mucosa surrounding them and possibly covering them in exudate [8].

Endoscopy-derived biopsies are essential for verification. Multinucleated giant cells with ground-glass nuclei and eosinophilic intranuclear inclusions (Cowdry type A inclusions) are the two main histological characteristics of HSV esophagitis. Usually seen at the ulcer boundaries, these characteristics point to HSV infection. Staining for HSV proteins with immunohistochemistry will help with diagnosis even more. This method more conclusively confirms the virus's presence by using antibodies specific to HSV antigens. Polymerase chain reaction (PCR) for HSV DNA may occasionally improve the precision of the diagnosis [9].

Other causes of esophageal ulcerations, such as cytomegalovirus (CMV) esophagitis, eosinophilic esophagitis, drug-induced esophagitis, and reflux esophagitis, are accounted for in the differential diagnosis of HSV esophagitis. For instance, immunocompromised patients may present similarly with CMV esophagitis; however, endoscopy usually reveals larger, more superficial ulcers. Instead of distinct ulcers, eosinophilic esophagitis may manifest as linear furrows or concentric rings. Lesions localized to specific locations where the pill may have stuck are common symptoms of drug-induced esophagitis in patients who have just started taking medication. Making the right diagnosis requires understanding these differences in clinical presentation, particularly when endoscopic and histological results coincide. Histopathology, immunohistochemistry, and PCR tests are used to confirm clinical suspicion in the diagnosis of HSV esophagitis, in accordance with established recommendations [10]. 

Treatment guidelines recommend starting antiviral therapy promptly with acyclovir, with valacyclovir or famciclovir as alternatives, particularly for patients who have difficulty with acyclovir’s dosing schedule. Monitoring during therapy should include assessment of renal function, especially in elderly patients, as acyclovir can cause nephrotoxicity. Preventative measures for vulnerable individuals, particularly the elderly, involve minimizing risk factors such as the use of immunosuppressive medications and managing comorbid conditions that could predispose to viral reactivation. Although HSV esophagitis in immunocompetent patients usually goes away in one to two weeks, antiviral medication is frequently used to hasten symptom resolution and avoid problems. Acyclovir is the recommended antiviral medication for HSV esophagitis. It prevents the synthesis of viral DNA, lowers viral replication, and speeds up the resolution of symptoms. The typical dosage of Acyclovir is 200 mg to 400 mg for immunocompetent patients, taken orally five times daily for seven to 10 days. In contrast, the dosage is increased from 400 mg to 800 mg orally five times daily for immunocompromised patients. Alternative antiviral medications with easier dosing schedules and improved oral absorption include valacyclovir and famciclovir. It is crucial to provide supportive care, which includes enough intravenous fluids, pain relief, and nutritional assistance. Analgesics and intravenous fluids may be necessary if oral intake is compromised. Maintaining patient strength and promoting healing can be achieved by ensuring appropriate nutritional intake with soft or liquid diets [11].

It is unclear exactly what mechanism causes HSV esophagitis in immunocompetent people. On the contrary, temporary immunosuppression is thought to be implicated. The host's immune system may be momentarily weakened by variables including physical or mental stress, coexisting illnesses, or brief use of immunosuppressive drugs (such as corticosteroids), which could lead to HSV reactivation and esophageal mucosal infection. To precisely identify the variables causing HSV esophagitis in immunocompetent patients, more investigation is required. Research examining the influence of various HSV strains on clinical manifestation, genetic susceptibility, and temporary immunosuppression is crucial. Furthermore, investigating the safety and effectiveness of more recent antiviral medications or combination therapy may offer better therapeutic alternatives [12].

Despite being rare in immunocompetent people, it can be difficult to diagnose because its symptoms can be confused with the symptoms of other esophageal disorders. Histopathological confirmation and endoscopic examination continue to be the mainstays of diagnosis. Antiviral therapy is frequently utilized in clinical practice since it is beneficial in speeding up the resolution of symptoms, especially when combined with acyclovir. More research is necessary into the possible association between HSV esophagitis and temporary immunosuppression to understand the pathophysiology better and develop management approaches. Supportive care and effective treatment can improve patient outcomes and quality of life [13].

## Conclusions

This case highlights how crucial it is to rule out HSV esophagitis when making a differential diagnosis for esophageal ulcers, especially in immunocompetent individuals. Early detection and antiviral medication treatment can reduce symptoms quickly. Prompt diagnosis relies on key investigations such as endoscopy with biopsy, histopathological examination, and PCR testing for HSV DNA, which are essential in confirming the presence of the virus and differentiating it from other causes of esophagitis. Healthcare professionals must be on surveillance for immunosuppressive variables that could put immunocompetent patients at risk for HSV infections, such as temporary immunosuppression. This case report aims to add to the increasing pool of knowledge on HSV esophagitis by offering insightful information on the pathophysiology, difficulties in diagnosing the condition, and treatment options for immunocompetent patients.
